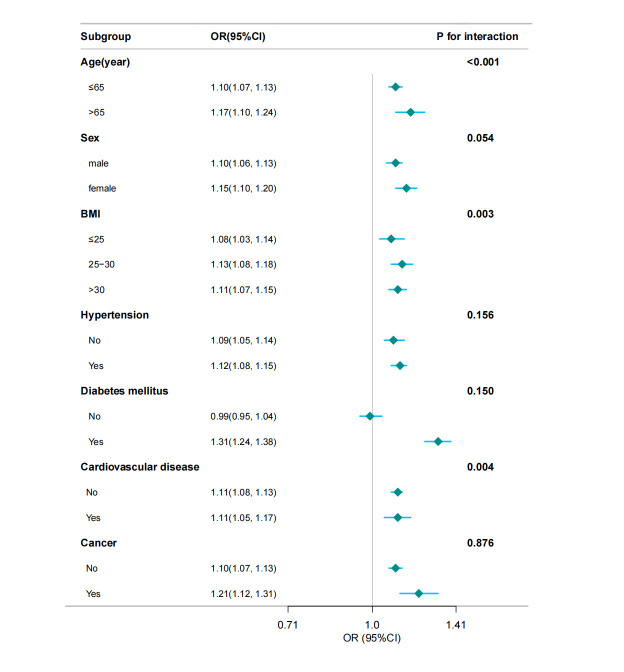# Association between the Zhejiang University index and biological aging in US adults: A cross-sectional study based on NHANES 1999 to 2018

**DOI:** 10.1097/MD.0000000000049205

**Published:** 2026-06-12

**Authors:** Chuantie Chen, Yuanyuan Chen, Guiyun Gao, Weiying Liu, Jinmin Cao

**Affiliations:** a Department of Gastroenterology, Longgang Central Hospital of Shenzhen, Shenzhen, Guangdong Province, China; b Department of Liver Diseases, Shenzhen Third People’s Hospital, Shenzhen, Guangdong Province, China; c Department of Infectious Diseases, The Second Clinical Medical College of Jinan University (Shenzhen People’s Hospital), Shenzhen, Guangdong Province, China; d Department of Dermatology, Hunan Aerospace Hospital, Changsha, Hunan Province, China.

**Keywords:** accelerated aging, biological age, biological aging, Klemera–Doubal method, NHANES, phenotypic age, ZJU index

## Abstract

Biological aging has garnered increasing attention. Although the Zhejiang University (ZJU) index was developed to predict fatty liver disease, a condition associated with biological aging, direct evidence connecting it to biological aging remains limited. This study aimed to investigate the relationship between the ZJU index and biological aging. This retrospective cross-sectional study analyzed data from 1999 to 2018, utilizing nationally representative data from the National Health and Nutrition Examination Survey. Weighted multivariable linear and logistic regression models were used to assess the associations between the ZJU index and biological aging, adjusting for age, sex, race, education level, marital status, income-to-poverty ratio, body mass index, smoking and drinking status, physical activity, hypertension, diabetes mellitus, cardiovascular disease, and cancer. Restricted cubic spline regression assessed nonlinear relationships, along with trend tests and subgroup analyses to ensure the robustness of the results. A total of 12,486 participants were enrolled in the study, with a mean age of 46.70 ± 0.28 years, and 49.76% were male. The ZJU index showed significant positive associations with both the Klemera–Doubal method biological age (adjusted β = 0.42, 95% confidence interval: 0.37, 0.46) and accelerated aging risk (adjusted odds ratio = 1.20, 95% confidence interval: 1.17, 1.24). Similarly, a 1-unit increase in the ZJU index was linked to a 0.60-year rise in phenotypic age and a 10% increase in the accelerated aging risk. Restricted cubic spline analyses revealed a linear relationship between the ZJU index and Klemera–Doubal method biological age (*P* for nonlinearity = .804), but nonlinear associations with phenotypic age and both age acceleration measures (all *P* for nonlinearity < .05). The ZJU index showed a positive association with biological aging across most subgroups. Higher levels of the ZJU index were correlated with greater biological age and a higher likelihood of accelerated aging.

## 1. Introduction

As the global population increasingly ages, the complex biological process of aging, characterized by progressive physiological deterioration and heightened susceptibility to disease,^[1]^ has become a key focus of current public health research. Unlike chronological age, which merely reflects the passage of time, biological age provides a more accurate prediction of an individual’s health risks and disease susceptibility by assessing the degree of physiological functional decline.^[2]^ For this reason, it has become an important indicator for health interventions and prognostic assessments.^[3]^ To quantify biological aging, a variety of methods have been proposed. One approach includes epigenetic clock algorithms based on deoxyribonucleic acid methylation data.^[4]^ Another set of methods involves predictive models derived from clinical biochemical markers, such as the Klemera–Doubal method (KDM) biological age,^[5]^ phenotypic age,^[6]^ homeostatic dysregulation,^[7]^ and allostatic load,^[8]^ their discrepancy with chronological age defined as biological age acceleration.^[9]^ These clinically based algorithms are as accurate as or more accurate than deoxyribonucleic acid methylation measures in predicting disease, disability, and mortality.^[10]^ They are sensitive to various factors hypothesized to accelerate aging.^[11,12]^ These methods provide strong support for assessing and validating the effects of antiaging interventions on the rate of aging in individuals within a relatively short timeframe.

The Zhejiang University (ZJU) index, developed by Zhejiang University,^[13]^ is a novel metabolic parameter constructed based on body mass index (BMI), fasting plasma glucose, triglycerides (TG), and the ratio of serum alanine aminotransferase to serum aspartate aminotransferase. Compared with individual biochemical markers, the ZJU index demonstrates significant advantages in diagnosing nonalcoholic fatty liver disease (NAFLD), providing a more comprehensive assessment of metabolic disorders and associated disease risks. Initially developed to predict NAFLD, it has been validated to possess robust predictive capabilities.^[14]^ Moreover, the ZJU index exhibits greater sensitivity and specificity than the Fatty Liver Index, Lipid Accumulation Product Index, Hepatic Steatosis Index, and Visceral Adiposity Index.^[15]^ Beyond its utility in NAFLD detection, the ZJU index captures key metabolic and inflammatory components, including insulin resistance, dyslipidemia, and hepatic injury, that are central to the pathophysiology of biological aging.^[16]^ While NAFLD itself has been associated with accelerated aging,^[17]^ the ZJU index may serve as a broader indicator of metabolic dysregulation that extends beyond liver-specific pathology. While the ZJU index has been validated as a robust predictor of NAFLD and associated metabolic disorders, its relationship with biological aging has not been examined. Specifically, no large-scale, population-based study has investigated whether the ZJU index is associated with KDM biological age or phenotypic age in a nationally representative US adult population. Furthermore, existing research on NAFLD and aging has primarily focused on clinical outcomes (e.g., mortality, disease progression) rather than on composite metabolic indices such as the ZJU index, which could serve as early, noninvasive biomarkers of accelerated aging. This study aims to fill these gaps.

This study investigates the relationship between the ZJU index and biological aging using a nationally representative sample from the National Health and Nutrition Examination Survey (NHANES) from 1999 to 2018. We hypothesized that a higher ZJU index would be positively associated with biological aging and accelerated aging, independent of demographic and lifestyle factors. This study aims to test this hypothesis in a nationally representative US sample, evaluate the dose-response relationship between the ZJU index and biological aging measures, and identify potential population subgroups where this association may be particularly pronounced.

## 2. Materials and methods

### 2.1. Study design and population

The NHANES was a national program designed to assess the health and nutritional status of the US population. It employs a rigorous multi-stage probabilistic sampling method to ensure national representativeness. The study protocol was approved by the Centers for Disease Control and Prevention’s National Center for Health Statistics research ethics review board. Participants provided written informed consent upon enrollment. This study utilized publicly accessible deidentified data; thus, ethical approval and consent were not necessary. Our secondary analysis adheres to the Strengthening the Reporting of Observational Studies in Epidemiology guidelines for cross-sectional studies. Comprehensive details about the NHANES survey can be accessed publicly at https://www.cdc.gov/nchs/nhanes.

**Figure 1.**
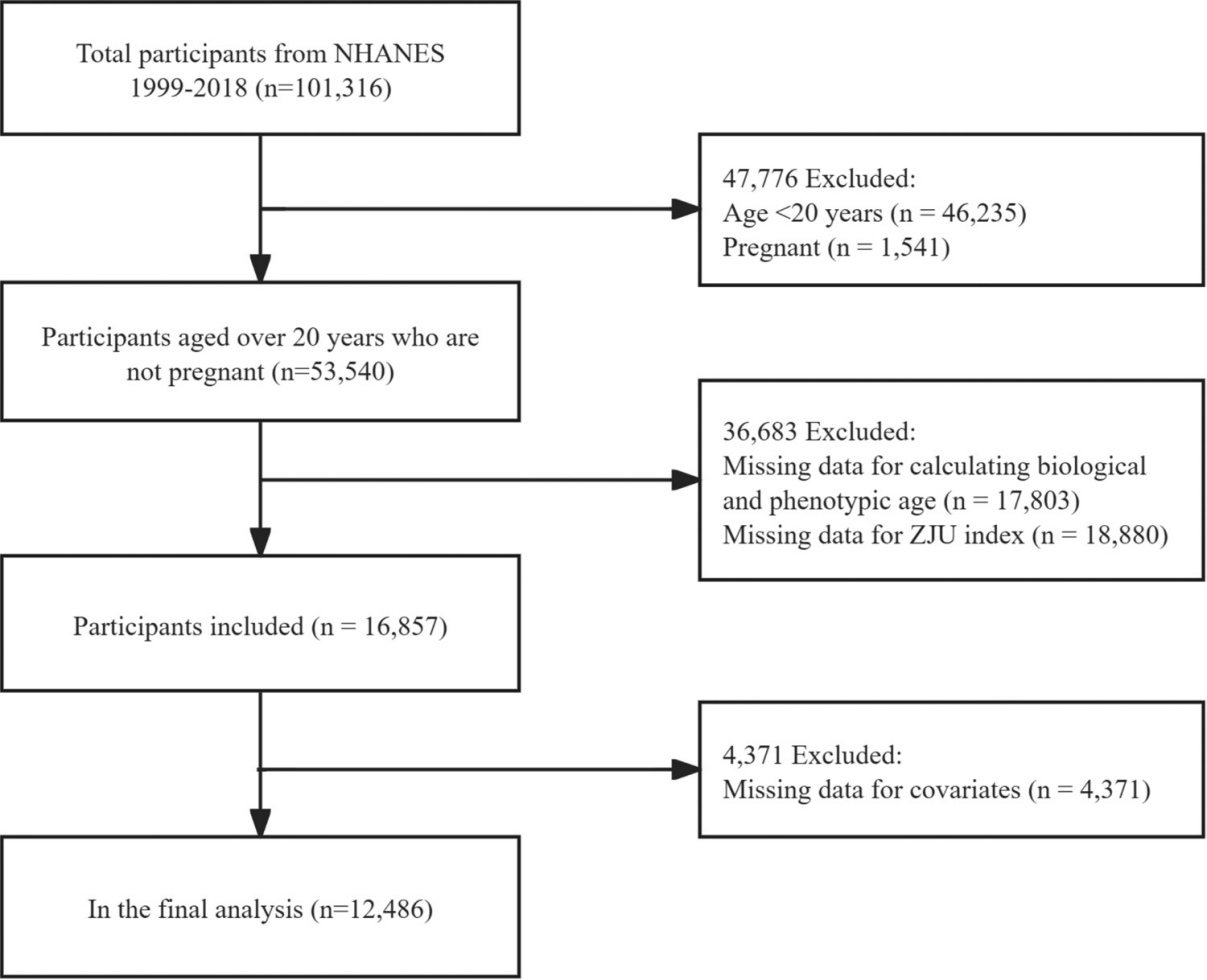
Flowchart of the participant selection. NHANES = National Health and Nutrition Examination Survey, ZJU index = Zhejiang University index.

Our analyses included 101,316 participants from the 1999 to 2018 NHANES cycles. Our exclusion criteria were as follows: age under 20 years (n = 46,235), pregnancy (n = 1541), missing data for calculating biological and phenotypic age (n = 17,803), missing data for the ZJU index (n = 18,880), and missing data for covariates (n = 4371). Ultimately, the study comprised 12,486 participants. Figure 1 depicts the screening process.

### 2.2. Definition of ZJU index

The ZJU index is calculated using the formula^[18]^:


$$\begin{array}{llll} ZJU\;index= & \;BMI\;\left(kg/m^{2}\right)+TG\;\left(mmol/L\right)\; & +ALT\;\left(IU/L\right)/AST\left(IU/L\right)\;\;ratio\;\times \;3\; & +FPG\;\left(mmol/L\right)\;\left(+2\;if\;females\right). \end{array}$$

### 2.3. Assessment of biological aging

In our study, biological age was assessed using the KDM biological age and phenotypic age.^[19]^ The KDM biological age prediction indicates an individual’s age under normal physiological conditions. This is determined by regressing clinical indicators, including systolic blood pressure, albumin, glycated hemoglobin, blood urea nitrogen, total cholesterol, creatinine, C-reactive protein, and alkaline phosphatase. Phenotypic age is calculated through a multivariate mortality hazard model utilizing clinical indicators such as chronological age, creatinine, glucose, albumin, C-reactive protein, mean cell volume, lymphocyte percentage, red cell distribution width, white blood cell count, and alkaline phosphatase. KDM biological age acceleration occurs when KDM biological age exceeds chronological age, and phenotypic age acceleration occurs when phenotypic age exceeds chronological age.

### 2.4. Covariates

Demographic data were collected to evaluate sociodemographic characteristics, including sex (male or female), age (years), race (non-Hispanic White, non-Hispanic Black, Mexican American, or Other), marital status (married, living alone, never married), education level (below high school, high school, or above high school), and income-to-poverty ratio (PIR) categorized as ≤1.3, >1.3 to 3.5, or >3.5. Physical activity was quantified using the Metabolic Equivalent of Task, and smoking and drinking statuses were categorized as never, former, or current. BMI data were derived from examination results and calculated as weight (kg) divided by the square of height (m^2^), then categorized into ≤25, 25 to 30, or >30. Hypertension was diagnosed based on self-reported history, current antihypertensive medication use, or an average systolic blood pressure of at least 140 mm Hg and/or diastolic pressure of at least 90 mm Hg. Diabetes mellitus was identified through self-reported physician diagnosis, fasting glucose levels of at least 7.0 mmol/L, glycated hemoglobin levels of 6.5% or higher, or the use of diabetes medication. Cancer and cardiovascular disease, such as coronary heart disease, heart failure, angina, stroke, and heart attack, were identified based on questionnaire responses regarding prior physician diagnoses.

### 2.5. Statistical analysis

Per the NHANES analysis guidelines, we used sample weighting codes “WTSAF4YR” and “WTSAF2YR” for statistical analysis. For 1999 to 2000, 2001 to 2002 year cycle, the sampling weights were calculated as 2/10 × WTSAF4YR, and 1/10 × WTSAF2YR for the remaining cycles. Analyses were conducted using complete case data. Baseline characteristics were divided into 3 groups according to the ZJU index levels. Continuous variables are presented as mean (standard error, SE). Categorical variables are displayed as numbers (percentages). We employed the chi-square test for categorical variables, one-way analysis of variance for normally distributed continuous variables, and the Kruskal–Wallis test for skewed continuous variables. Weighted multivariable linear and logistic regression models were used to assess associations between the ZJU index and KDM biological/phenotypic age, as well as their age accelerations, estimating β coefficients (linear) or odds ratios with 95% confidence intervals. The crude model was unadjusted. Model 1 was adjusted for age and sex. Model 2 was further adjusted for race, education level, marital status, and PIR based on Model 1. Model 3 was further adjusted for BMI, smoking and drinking status, physical activity, diabetes mellitus, cardiovascular disease, hypertension, and cancer based on Model 2. A restricted cubic spline with 3 knots was adopted to visualize the potentially nonlinear association between ZJU index levels and KDM biological/phenotypic age and their accelerations, after adjustment for Model 3 variables. A two-piecewise linear/logistic regression model was developed to identify potential threshold effects after adjustment for Model 3 variables. Subgroup analyses were conducted across categories of age, sex, BMI, diabetes mellitus, hypertension, cardiovascular disease, and cancer, with interactions examined. Statistical analyses were conducted using R software (v4.2.2; The R Foundation, https://www.R-project.org) and the Free Statistics analysis platform (v2.1.1; Beijing Free Clinical Medical Technology Co., Ltd., https://www.clinicalscientists.cn/freestatistics/). A two-tailed *P* < .05 indicated statistical significance.

## 3. Results

### 3.1. Baseline characteristics of participants

Table 1 presents the demographic, comorbidity, and socioeconomic data for the 12,486 participants, representing 120,622,760 US adults. The study included 6401 males (49.76%), with a mean age of 46.70 ± 0.28 years. Participants were divided into 4 groups based on ZJU index quartiles: Q1 < 34.69, Q2 (34.69–39.11), Q3 (39.12–44.42), and Q4 ≥ 44.42. Participants in the highest ZJU index quartile were older, predominantly female, and more likely to be non-Hispanic White compared with those in the lowest quartile. They had lower education levels, lower PIR, engaged in less physical activity, and were less likely to smoke or consume alcohol. Additionally, they exhibited higher BMI and increased rates of hypertension, diabetes mellitus, and cardiovascular disease, with no observed differences in cancer prevalence. Participants in the Q4 group had higher phenotypic and biological ages, as well as increased KDM biological age and phenotypic age acceleration.

**Table 1:** Baseline characteristics of participants by quartiles of Zhejiang University index.

Characteristics	Total	ZJU index quartiles				*P*-value
		Q1 < 34.69	Q2 (34.69–39.11)	Q3 (39.12–44.42)	Q4 ≥ 44.42	
Sample size, N, weighted	120,622,760	33,206,630	30,204,532	29,073,582	28,138,016	
Age, yr	46.70 (0.28)	42.70 (0.44)	47.71 (0.44)	49.09 (0.42)	47.85 (0.34)	<.001
Sex, n (unweighted) (%)						<.001
Male	6401 (49.76%)	1720 (48.98%)	1735 (54.12%)	1646 (52.84%)	1300 (42.82%)	
Female	6085 (50.24%)	1402 (51.02%)	1386 (45.88%)	1475 (47.16%)	1822 (57.18%)	
Race, n (unweighted) (%)						<.001
Non-Hispanic White	2422 (7.48%)	381 (4.91%)	594 (7.31%)	730 (9.27%)	717 (8.86%)	
Non-Hispanic Black	910 (4.66%)	179 (3.52%)	250 (5.25%)	255 (5.45%)	226 (4.56%)	
Mexican American	6229 (72.32%)	1751 (74.82%)	1627 (73.47%)	1466 (70.63%)	1385 (69.88%)	
Other	2925 (15.54%)	811 (16.75%)	650 (13.96%)	670 (14.65%)	794 (16.71%)	
Marital status, n (unweighted) (%)						<.001
Married	7774 (66.31%)	1782 (61.30%)	2016 (67.74%)	2037 (70.09%)	1939 (66.80%)	
Living alone	2759 (17.40%)	608 (15.46%)	667 (17.42%)	718 (17.68%)	766 (19.37%)	
Never married	1953 (16.29%)	732 (23.25%)	438 (14.84%)	366 (12.24%)	417 (13.83%)	
Education levels, n (unweighted) (%)						<.001
Below high school	3497 (17.37%)	744 (15.14%)	827 (16.14%)	942 (18.77%)	984 (19.89%)	
High school	2962 (24.96%)	689 (22.10%)	710 (23.70%)	814 (28.53%)	749 (25.99%)	
Above high school	6027 (57.67%)	1689 (62.76%)	1584 (60.16%)	1365 (52.70%)	1389 (54.13%)	
PIR, n (unweighted) (%)						<.001
≤1.30	3529 (19.12%)	864 (19.05%)	808 (16.54%)	884 (19.27%)	973 (21.81%)	
1.30–3.50	4924 (37.17%)	1204 (36.70%)	1208 (35.58%)	1255 (37.71%)	1257 (38.87%)	
>3.50	4033 (43.71%)	1054 (44.25%)	1105 (47.88%)	982 (43.02%)	892 (39.32%)	
BMI, kg/m^2^	28.52 (0.09)	22.00 (0.05)	26.21 (0.04)	29.90 (0.05)	37.28 (0.15)	<.001
Smoking status, (%)						<.001
Never	6422 (51.30%)	1544 (50.17%)	1611 (51.83%)	1631 (51.64%)	1636 (51.70%)	
Former	3357 (26.07%)	675 (20.85%)	874 (26.73%)	911 (28.43%)	897 (29.06%)	
Now	2707 (22.64%)	903 (28.98%)	636 (21.43%)	579 (19.93%)	589 (19.24%)	
Drinking status, (%)						<.001
Never	1714 (11.20%)	387 (10.81%)	391 (9.66%)	421 (10.95%)	515 (13.56%)	
Former	1932 (13.68%)	377 (10.50%)	448 (13.18%)	496 (14.17%)	611 (17.45%)	
Now	8840 (75.13%)	2358 (78.69%)	2282 (77.16%)	2204 (74.88%)	1996 (68.99%)	
Physical activity, MET-min/week	2200.04 (69.85)	2365.98 (137.68)	2334.36 (138.14)	2064.74 (96.44)	1999.82 (100.48)	<.001
Hypertension, n (unweighted) (%)						<.001
No	5698 (51.74%)	1942 (69.66%)	1534 (54.83%)	1282 (45.85%)	940 (33.35%)	
Yes	6788 (48.26%)	1180 (30.34%)	1587 (45.17%)	1839 (54.15%)	2182 (66.65%)	
Diabetes mellitus (%)						<.001
No	10,391 (88.10%)	3007 (97.61%)	2833 (93.72%)	2584 (88.06%)	1967 (70.89%)	
Yes	2095 (11.90%)	115 (2.39%)	288 (6.28%)	537 (11.94%)	1155 (29.11%)	
Cardiovascular disease (%)						<.001
No	11,085 (91.73%)	2890 (94.92%)	2778 (91.91%)	2732 (90.15%)	2685 (89.41%)	
Yes	1401 (8.27%)	232 (5.08%)	343 (8.09%)	389 (9.85%)	437 (10.59%)	
Cancer (%)						.390
No	11,311 (91.24%)	2843 (92.03%)	2795 (91.04%)	2834 (90.67%)	2839 (91.11%)	
Yes	1175 (8.76%)	279 (7.97%)	326 (8.96%)	287 (9.33%)	283 (8.89%)	
KDM biological age, yr	46.40 (0.28)	40.99 (0.43)	46.84 (0.41)	48.93 (0.40)	49.68 (0.32)	<.001
Phenotypic age, yr	42.85 (0.33)	36.42 (0.49)	42.44 (0.47)	45.35 (0.44)	48.32 (0.42)	<.001
KDM biological age acceleration, (%)						<.001
No	6888 (55.16%)	2138 (69.86%)	1903 (60.02%)	1683 (52.80%)	1164 (35.06%)	
Yes	5598 (44.84%)	984 (30.14%)	1218 (39.98%)	1438 (47.20%)	1958 (64.94%)	
Phenotypic age acceleration, (%)						<.001
No	9232 (77.56%)	2637 (88.03%)	2579 (85.00%)	2348 (77.82%)	1668 (56.94%)	
Yes	3254 (22.44%)	485 (11.97%)	542 (15.00%)	773 (22.18%)	1454 (43.06%)	

BMI = body mass index, KDM = Klemera–Doubal method, MET = metabolic equivalent of task, PIR = income-to-poverty ratio, Q = quartile, ZJU index = Zhejiang University index.

### 3.2. Association between ZJU index and biological aging

**Figure 2.**
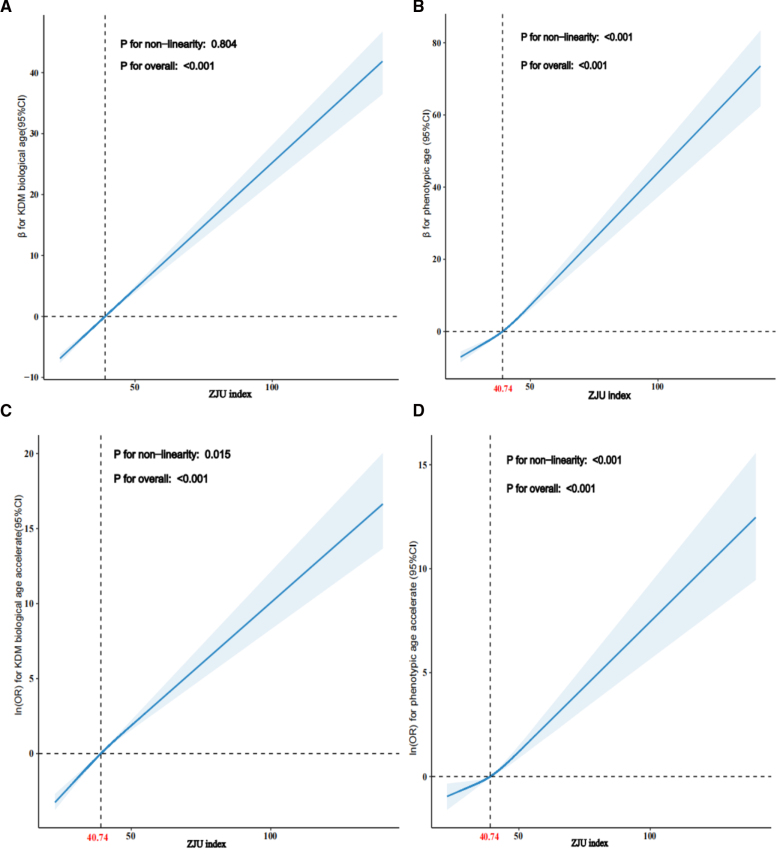
Association of Zhejiang University index with Klemera–Doubal method biological age/phenotypic age (A, B) and Klemera–Doubal method biological age/phenotypic age acceleration (C, D). Data were analyzed using multivariable linear/logistic regression with restricted cubic splines. Models were adjusted for age, sex, race, education levels, marital status, PIR, BMI, smoking status, drinking status, physical activity, hypertension, diabetes mellitus, cardiovascular disease, and cancer. β = beta, BMI = body mass index, CI = confidence interval, KDM = Klemera–Doubal method, OR = odds ratio, PIR = income-to-poverty ratio, ZJU index = Zhejiang University index.

Survey-weighted multivariable linear regression revealed a significant positive association between the ZJU index and KDM biological age/phenotypic age. In the crude model, each unit increase in the ZJU index was associated with a 0.4-year increase in KDM biological age and an 8% higher likelihood of accelerated aging (Tables 2 and 3). The positive association remained robust after adjustment in Model 3. Specifically, a 1-unit increase in the ZJU index was linked to a 0.42-year rise in KDM biological age and a 20% greater risk of accelerated aging (Tables 2 and 3). Similarly, a 1-unit increase in the ZJU index corresponded to a 0.60-year increase in phenotypic age, with a 10% increased risk of accelerated aging (Tables 2 and 3). When the ZJU index was divided into quartiles (with Q1 as the reference), the Q4 group demonstrated a 2.32-year increase in KDM biological age and a 2.06-year increase in phenotypic age (Table 2). Moreover, the Q4 group had a 241% higher risk of accelerated aging via KDM biological age acceleration and a 41% higher risk via phenotypic age acceleration (Table 3). The restricted cubic spline analysis confirmed a linear relationship between the ZJU index and KDM biological age (*P* for nonlinearity = .804), whereas a nonlinear association was found between the ZJU index and KDM biological age acceleration (*P* for nonlinearity = .015; Fig. 2A and B). In contrast, both phenotypic age and phenotypic age acceleration showed nonlinear associations (*P* for nonlinearity < .001 for both; Fig. 2C and D), with a turning point at 40.74 (Tables S1 and S2, Supplemental Digital Content). For ZJU index values exceeding 40.74, each 1-unit increase was associated with a significant rise in both phenotypic age and phenotypic age acceleration. These effect sizes are clinically meaningful, indicating that the observed associations are not only statistically significant but also of practical importance.

**Table 2:** Association between Zhejiang University index and Klemera–Doubal method biological age/phenotypic age.

	Crude model		Model 1		Model 2		Model 3	
	β (95% CI)	*P*-value	β (95% CI)	*P*-value	β (95% CI)	*P*-value	β (95% CI)	*P*-value
KDM biological age								
ZJU index (continuous)	0.40 (0.35, 0.45)	<.001	0.21 (0.19, 0.22)	<.001	0.21 (0.19, 0.22)	<.001	0.42 (0.37, 0.46)	<.001
ZJU index quartile								
Q1 < 34.69	Reference		Reference		Reference		Reference	
Q2 (34.69–39.11)	5.85 (4.87, 6.83)	<.001	1.08 (0.84, 1.32)	<.001	1.13 (0.89, 1.37)	<.001	0.78 (0.55, 1.01)	<.001
Q3 (39.12–44.42)	7.94 (7.05, 8.83)	<.001	1.91 (1.69, 2.13)	<.001	1.92 (1.70, 2.14)	<.001	1.22 (0.95, 1.49)	<.001
Q4 ≥ 44.42	8.69 (7.75, 9.62)	<.001	3.94 (3.67, 4.21)	<.001	3.93 (3.66, 4.20)	<.001	2.32 (1.88, 2.76)	<.001
*P* for trend		<.001		<.001		<.001		<.001
Phenotypic age								
ZJU index (continuous)	0.59 (0.54, 0.64)	<.001	0.38 (0.35, 0.41)	<.001	0.37 (0.34, 0.40)	<.001	0.60 (0.50, 0.70)	<.001
ZJU index quartile								
Q1 < 34.69	Reference		Reference		Reference		Reference	
Q2 (34.69–39.11)	6.02 (4.94, 7.10)	<.001	0.69 (0.28, 1.09)	.001	0.80 (0.39, 1.20)	<.001	−0.07 (−0.49, 0.35)	.736
Q3 (39.12–44.42)	8.94 (7.85, 10.02)	<.001	2.19 (1.75, 2.64)	<.001	2.20 (1.77, 2.63)	<.001	0.33 (−0.20, 0.85)	.219
Q4 ≥ 44.42	11.91 (10.82, 12.99)	<.001	6.65 (6.04, 7.25)	<.001	6.56 (5.98, 7.15)	<.001	2.06 (1.14, 2.97)	<.001
*P* for trend		<.001		<.001		<.001		.001

Crude model: no other covariates were adjusted.

Model 1: adjusted for age and sex.

Model 2: adjusted for age, sex, race, education, marital status, and PIR.

Model 3: adjusted for age, sex, race, education levels, marital status, PIR, BMI, smoking status, drinking status, physical activity, hypertension, diabetes mellitus, cardiovascular disease, and cancer.

β = beta, BMI = body mass index, CI = confidence interval, KDM = Klemera–Doubal method, PIR = income-to-poverty ratio, Q = quartile, ZJU index = Zhejiang University index.

**Table 3:** Association between Zhejiang University index and Klemera–Doubal method biological age/phenotypic age acceleration.

	Crude model		Model 1		Model 2		Model 3	
	OR (95% CI)	*P*-value	OR (95% CI)	*P*-value	OR (95% CI)	*P*-value	OR (95% CI)	*P*-value
KDM biological age acceleration								
ZJU index (continuous)	1.08 (1.07, 1.09)	<.001	1.10 (1.09, 1.11)	<.001	1.10 (1.09, 1.11)	<.001	1.20 (1.17, 1.24)	<.001
ZJU index quartile								
Q1 < 34.69	Reference		Reference		Reference		Reference	
Q2 (34.69–39.11)	1.54 (1.35, 1.77)	<.001	1.84 (1.60, 2.12)	<.001	1.93 (1.68, 2.22)	<.001	1.70 (1.45, 1.99)	<.001
Q3 (39.12–44.42)	2.07 (1.81, 2.37)	<.001	2.70 (2.34, 3.11)	<.001	2.82 (2.45, 3.25)	<.001	2.15 (1.81, 2.57)	<.001
Q4 ≥ 44.42	4.29 (3.76, 4.91)	<.001	6.17 (5.37, 7.08)	<.001	6.37 (5.53, 7.34)	<.001	3.41 (2.55, 4.56)	<.001
*P* for trend		<.001		<.001		<.001		<.001
Phenotypic age acceleration								
ZJU index (continuous)	1.10 (1.09, 1.11)	<.001	1.11 (1.10, 1.12)	<.001	1.11 (1.10, 1.12)	<.001	1.10 (1.07, 1.13)	<.001
ZJU index quartile								
Q1 < 34.69	Reference		Reference		Reference		Reference	
Q2 (34.69–39.11)	1.3 (1.10, 1.53)	.002	1.18 (0.99, 1.40)	.058	1.24 (1.04, 1.47)	.017	0.90 (0.75, 1.07)	.231
Q3 (39.12–44.42)	2.1 (1.73, 2.53)	<.001	1.89 (1.56, 2.29)	<.001	1.97 (1.61, 2.41)	<.001	1.03 (0.83, 1.28)	.772
Q4 ≥ 44.42	5.56 (4.62, 6.69)	<.001	5.45 (4.52, 6.57)	<.001	5.73 (4.72, 6.95)	<.001	1.41 (1.05, 1.90)	.023
*P* for trend		<.001		<.001		<.001		.043

Crude model: no other covariates were adjusted.

Model 1: adjusted for age and sex.

Model 2: adjusted for age, sex, race, education, marital status, and PIR.

Model 3: adjusted for age, sex, race, education levels, marital status, PIR, BMI, smoking status, drinking status, physical activity, hypertension, diabetes mellitus, cardiovascular disease, and cancer.

BMI = body mass index, CI = confidence interval, KDM = Klemera–Doubal method, OR = odds ratio, PIR = income-to-poverty ratio, Q = quartile, ZJU index = Zhejiang University index.

### 3.3. Subgroup analyses of the ZJU index and biological aging

Subgroup analyses were conducted to evaluate the consistency of the association between the ZJU index and biological aging across different demographic and clinical strata, including age (≤65 or >65), sex (male or female), BMI (≤25, 25–30, or >30), hypertension, diabetes mellitus, cardiovascular disease, and cancer. The positive association between the ZJU index and biological aging was observed across most subgroups, with effect estimates in the positive direction. For KDM biological age (Fig. S1, left panel, Supplemental Digital Content): The positive association between the ZJU index and KDM biological age was generally consistent across most strata. Significant interaction effects were observed for sex (*P* < .001), age (*P* < .001), and diabetes mellitus (*P* = .004), with stronger associations in females, participants with diabetes, and those aged ≤65 years. No significant interactions were found for BMI (*P* = .412), hypertension (*P* = .050), cardiovascular disease (*P* = .450), or cancer (*P* = .395). For phenotypic age (Fig. S1, right panel, Supplemental Digital Content): Significant interactions were observed for sex (*P* = .014), age (*P* < .001), BMI (*P* < .001), cardiovascular disease (*P* = .013), and diabetes mellitus (*P* < .001), with stronger associations in females, participants with diabetes or cardiovascular disease, those aged ≤65 years, and those with BMI > 30. No significant interactions were found for hypertension (*P* = .841) or cancer (*P* = .287). For KDM biological age acceleration (Fig. S2, Supplemental Digital Content): Significant interaction effects were observed for age (*P* < .001), hypertension (*P* < .001), and cardiovascular disease (*P* = .009), with stronger associations in participants aged ≤65 years, those without hypertension, and those without cardiovascular disease. No significant interactions were found for sex (*P* = .149), BMI (*P* = .060), diabetes mellitus (*P* = .448), or cancer (*P* = .108). For phenotypic age acceleration (Fig. S3, Supplemental Digital Content): Significant interaction effects were observed for age (*P* < .001), BMI (*P* = .003), and cardiovascular disease (*P* = .004), with stronger associations in females, participants with cardiovascular disease, and those aged >65 years. No significant interactions were found for sex (*P* = .054), hypertension (*P* = .156), diabetes mellitus (*P* = .150), or cancer (*P* = .876).

## 4. Discussion

This national cross-sectional study identified consistent links between a higher ZJU index and accelerated biological aging, which remained significant after adjusting for covariates and irrespective of the definition of biological age. Trend tests and subgroup analyses confirmed the robustness of our findings, highlighting a positive relationship between the ZJU index and biological aging that remained consistent across all subgroups, even in the presence of significant interactions.

ZJU index, a novel metabolic-related biomarker, was initially developed for the diagnosis of NAFLD. The existing literature has predominantly focused on the associations between biological aging and clinical outcomes of NAFLD, such as the elevated risk of NAFLD onset, worsening of liver fibrosis,^[18]^ progression to nonalcoholic steatohepatitis,^[20]^ and increased risk of hepatocellular carcinoma.^[21]^ Consistent with these findings, a recent study demonstrated that the prevalence of NAFLD gradually increases with biological age.^[22,23]^ In addition, various aging markers (e.g., KDM age acceleration, phenotypic age acceleration) have been linked to NAFLD.^[24,25]^ These findings provide important insights into the interplay between metabolic disorders and the aging process and indirectly support the biological plausibility of the ZJU index as a potential marker of aging. It is important to note that evidence linking metabolic indices to biological aging is not entirely consistent. While the ZJU index demonstrated robust associations in our study, Huang et al^[26]^ reported that insulin resistance indices vary considerably in predictive value across populations. Additionally, Zhang et al^[27]^ showed that a healthy diet attenuates the biological age–NAFLD association, suggesting lifestyle factors may modify this relationship. These inconsistencies highlight the need for prospective, multi-ethnic studies to validate our findings. However, our study reveals an independent positive correlation between the ZJU index and biological aging, offering a significant contribution to this emerging field.

Notably, we observed significant interaction effects in gender-stratified analyses, with all odds ratios showing consistent directional associations, indicating robust positive correlations. Sex-specific differences were observed, with a stronger association between the ZJU index and biological aging in women, consistent with previous findings.^[28,29]^ This sexual dimorphism may be attributed to 2 key factors: first, women typically exhibit a gynoid (gluteofemoral) subcutaneous fat distribution, whereas men predominantly show android (visceral) fat accumulation.^[30]^ On the other hand, estrogen, prevalent in females, is associated with slower biological aging progression.^[31]^ Similarly, age-stratified analyses revealed significant differential effects, with stronger associations observed in younger populations (<65 years), aligning with multiple prior reports.^[13]^ Although chronological aging is irreversible, emerging evidence indicates that predicted biological age remains responsive to targeted interventions.^[32–34]^ Clinical observations suggest that lifestyle modifications, including dietary optimization and regular physical activity, may be associated with slowing the biological aging process.^[35]^

While the precise biological mechanisms underlying the association between the ZJU index and biological aging remain to be fully elucidated, several plausible links can be suggested from existing evidence. First, an elevated ZJU index is associated with insulin resistance, obesity, and metabolic disorders,^[26,36]^ which have been linked to oxidative stress and cellular senescence. Second, a higher ZJU index correlates with elevated inflammatory markers, including C-reactive protein,^[37,38]^ which is well-established to be involved in biological aging processes. Additionally, the ZJU index is linked to age-related diseases such as cardiovascular events, metabolic syndrome, and type 2 diabetes,^[39]^ supporting its relevance to biological aging. These factors together may underlie the observed association.

This study had several limitations. First, the cross-sectional design precludes causal inference, and reverse causality cannot be ruled out. Second, although we adjusted for multiple confounders, residual confounding may still exist (likely leading to overestimation), while overadjustment is also a potential concern (possibly causing underestimation). Third, as only baseline biomarker data were available, we were unable to evaluate whether longitudinal changes in the ZJU index influence biological aging. Fourth, biomarker variability may introduce measurement error, as all biomarkers were assessed at a single time point, and the ZJU index was originally developed in a Chinese population. Additionally, the exclusion of participants with missing data may have introduced bias if missingness was not completely random. Despite these limitations, the large, nationally representative sample and rigorous statistical adjustments may help support the robustness of our findings.

This study demonstrates that elevated ZJU index values are significantly associated with advanced biological age and an increased risk of accelerated aging. These findings suggest that the ZJU index is associated with the risk of accelerated aging. Future prospective cohort studies and mechanistic investigations are warranted to validate these findings and further elucidate the underlying relationship between the ZJU index and biological aging. In conclusion, this study demonstrates a positive association between the ZJU index and biological aging in a nationally representative US adult population.

## Acknowledgments

We thank the staff at the National Center for Health Statistics of the Centers for Disease Control for designing, collecting, and collating the NHANES data and creating the public database.

## Author contributions

**Conceptualization:** Yuanyuan Chen.

**Methodology:** Chuantie Chen, Yuanyuan Chen.

**Data curation:** Guiyun Gao, Weiying Liu.

**Supervision:** Guiyun Gao, Weiying Liu.

**Formal analysis:** Jinmin Cao.

**Software:** Chuantie Chen, Jinmin Cao.

**Writing – original draft:** Chuantie Chen, Yuanyuan Chen.

**Writing – review & editing:** Chuantie Chen, Yuanyuan Chen, Jinmin Cao.





**Figure:**
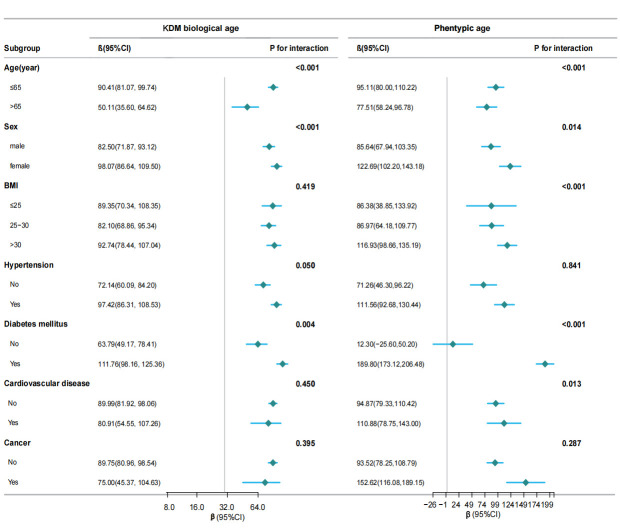


**Figure:**
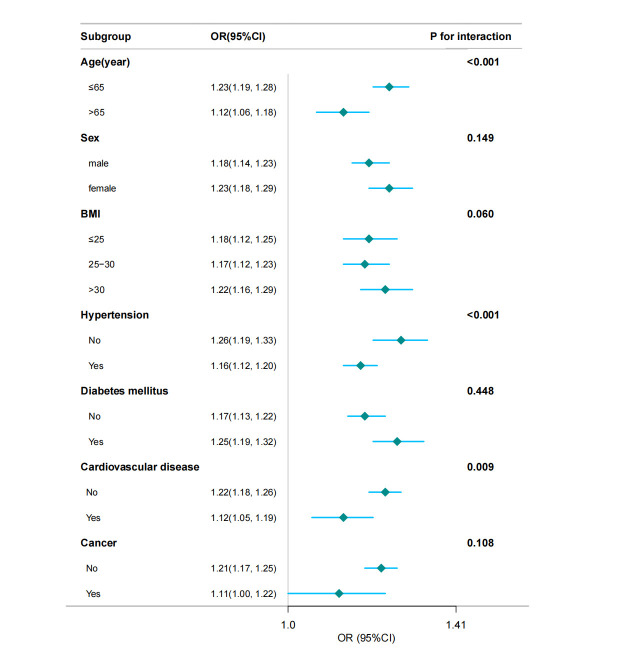


**Figure:**