# Supplementary material for: Association between the Zhejiang University index and biological aging in US adults: A cross-sectional study based on NHANES 1999 to 2018
Source: Medicine (Baltimore). 2026 Jun 12;105(24):e49205. doi: 10.1097/MD.0000000000049205 (PMC13268445; doi:10.1097/MD.0000000000049205)
Supplement: Supplementary file 2 [file medi-105-e49205-s005.docx]

**Table S2. Threshold effect analysis of the relationship of Zhejiang University index and phenotypic age acceleration**

| **ZJU index** | **Adjusted Model** | |
| --- | --- | --- |
|  | **OR (95%CI)** | ***P*-value** |
| <40.74 | 1.02 (0.96, 1.09) | 0.490 |
| ≥40.74 | 1.13 (1.10, 1.17) | <0.001 |
| Likelihood Ratio test |  | <0.001 |

Adjusted for age, sex, race, education levels, marital, PIR, BMI, smoking status, drinking status, physical activity, hypertension, diabetes mellitus, cardiovascular disease, cancer

ZJU index, Zhejiang University index; PIR, income to poverty ratio; BMI, body mass index; OR, odds ratio; CI, confidence interval
